# Supplementary figures and images for: Pre-imaginal conditioning alters adult sex pheromone response in Drosophila
Source: PeerJ. 2018 Sep 27;6:e5585. doi: 10.7717/peerj.5585 (PMC6164551; doi:10.7717/peerj.5585)

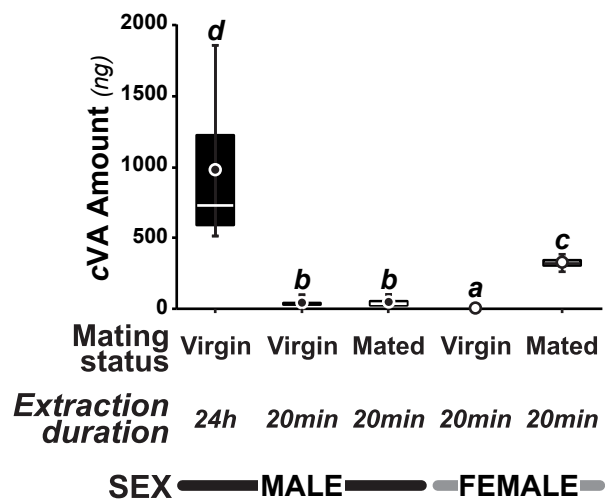

Supplement: Figure S1 — Box plots indicate the amount of cVA (ng/fly) detected on virgin and recently mated male and female flies (n = 9 − 17). We used solvent extraction over two periods: the 20 min-long extraction revealed the cVA present on the fly cuticle, whereas the 24 h-long solvent extraction (at 40 °C) allowed us to extract internal cVA. For more details see Fig. 3. [file peerj-06-5585-s001.pdf]

**A**

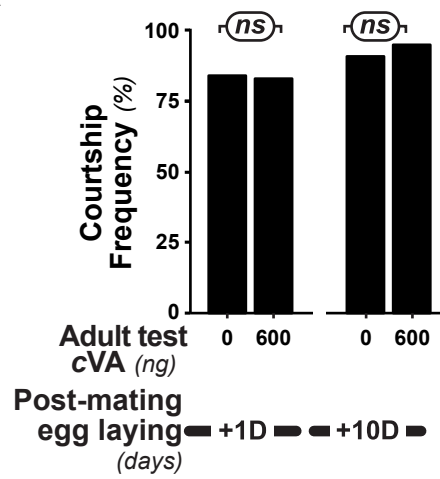

**B**

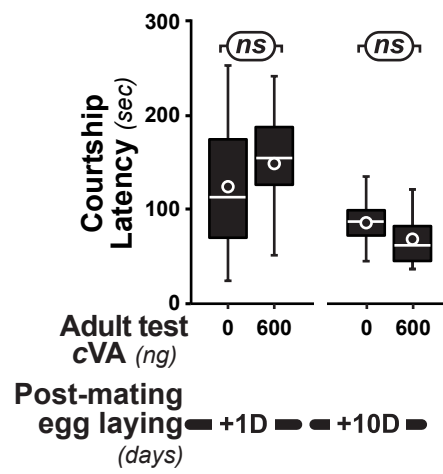

Supplement: Figure S2 — Courtship frequency (A) and latency (B) were measured in focal males derived from +1D and +10D eggs in the presence of 0 or 600 ng cVA. For more details see Figs. 1 and 4. [file peerj-06-5585-s002.pdf]

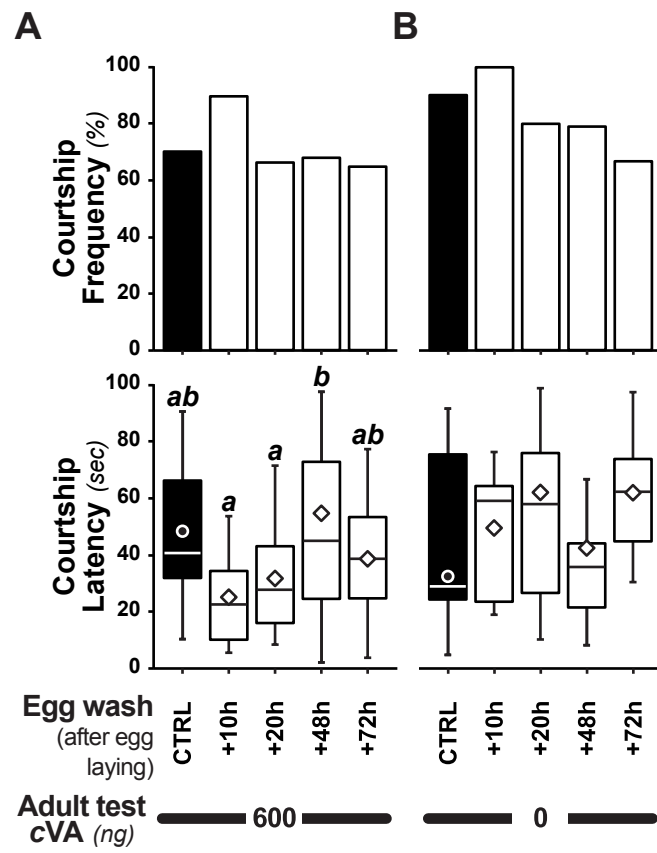

Supplement: Figure S3 — Courtship latency was measured in focal males derived from control eggs (filled bars) or from eggs washed at different times after egg-laying (AEL; +10, +20, +48, +72 h; empty bars) with 600 ng cVA (A) or no cVA (B). For more details see Figs. 1 and 5. [file peerj-06-5585-s003.pdf]

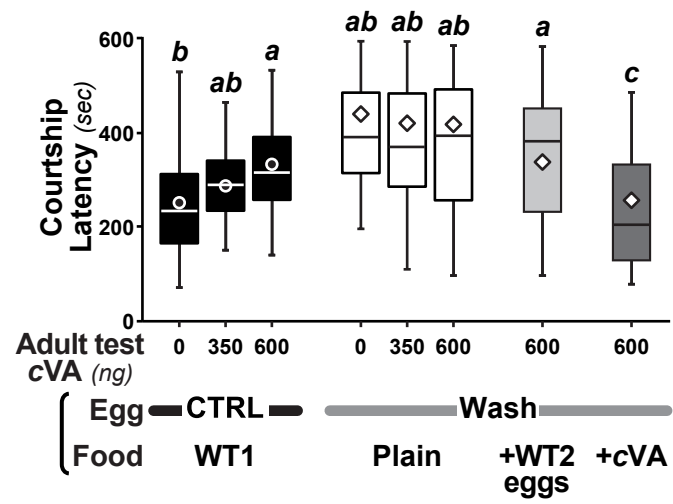

Supplement: Figure S4 — Courtship latency was measured in focal males each paired with a beheaded female in the presence of 0, 350 or 600 ng cVA. Focal males were derived from +1D eggs either with no treatment (filled bars), or from washed eggs raised either on plain food (empty bars), or on food seeded with WT2 eggs (light gray bars), or on cVA-rich food. For more details see Fig. 3. [file peerj-06-5585-s004.pdf]

**A**

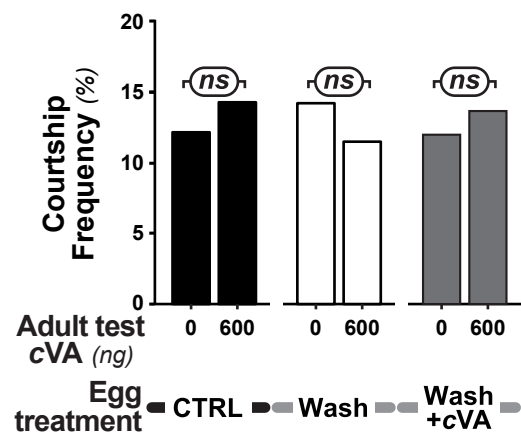

**B**

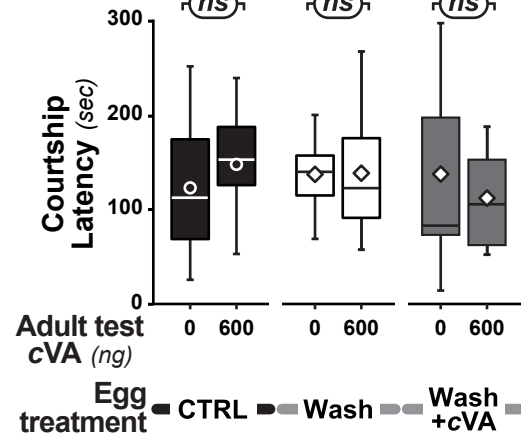

Supplement: Figure S5 — Courtship frequency (A) and latency (B) were measured in focal males dervived from control +1D eggs (filled bars), or of washed +1D eggs (empty bars), some of which were covered with a synthetic cVA solution (shaded bars). All eggs were placed on plain food and resulting males were tested with 0 or 600 ng cVA. For more details see Figs. 1 and 6. [file peerj-06-5585-s005.pdf]

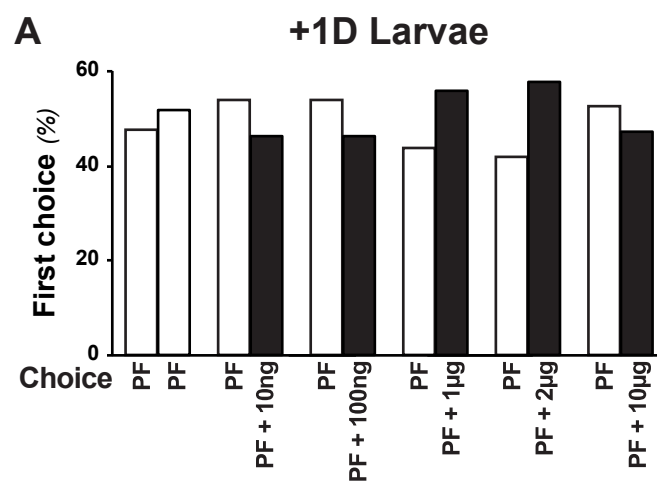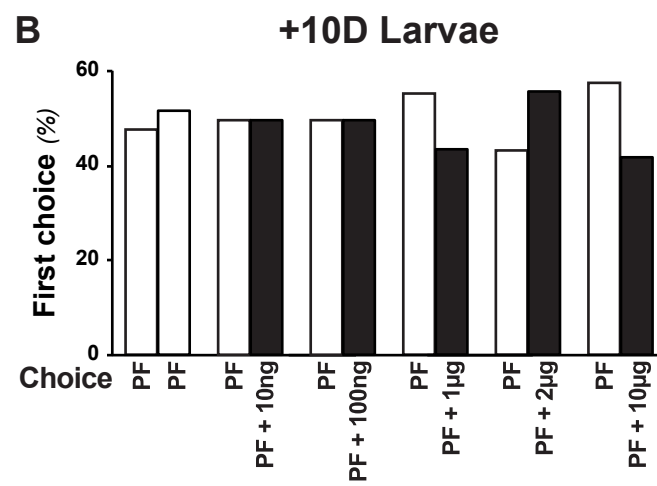

Supplement: Figure S6 — Individual larvae produced by +1D (A) or +10D (B) control eggs were placed on an agar plate with two food sources at opposite sides. These were either plain food (PF) or PF with the addition of 1 ng –10 μg of cVA. N = 50 for each comparison. The first food source reached by each larva was noted. There were no significant differences in response to cVA, nor were there any differences between +1D or +10D larvae. [file peerj-06-5585-s006.pdf]

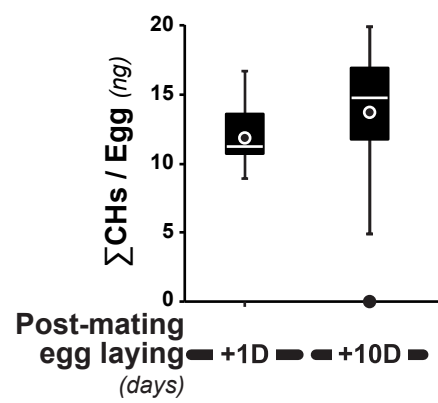

Supplement: Figure S7 — Comparison of total CHs amount on eggs laid by the females either one day (+1D) or 10 days (+10D) after mating (n = 10; 50 pooled eggs were extracted for each female). For more details, see Fig. 1. [file peerj-06-5585-s007.pdf]
